# Bioinformatic and systems biology approach revealing the shared genes and molecular mechanisms between COVID-19 and non-alcoholic hepatitis

**DOI:** 10.3389/fmolb.2023.1164220

**Published:** 2023-06-19

**Authors:** Huishuang Lu, Jiaxiu Ma, Yalan Li, Jin Zhang, Yaxin An, Wei Du, Xuefei Cai

**Affiliations:** The Key Laboratory of Molecular Biology of Infectious Diseases Designated by the Chinese Ministry of Education, Chongqing Medical University, Chongqing, China

**Keywords:** coronavirus disease 2019, non-alcoholic steatohepatitis, hub genes, network analysis, bioinformatics

## Abstract

**Introduction:** Coronavirus disease 2019 (COVID-19) has become a global pandemic and poses a serious threat to human health. Many studies have shown that pre-existing nonalcoholic steatohepatitis (NASH) can worsen the clinical symptoms in patients suffering from COVID-19. However, the potential molecular mechanisms between NASH and COVID-19 remain unclear. To this end, key molecules and pathways between COVID-19 and NASH were herein explored by bioinformatic analysis.

**Methods:** The common differentially expressed genes (DEGs) between NASH and COVID-19 were obtained by differential gene analysis. Enrichment analysis and protein-protein interaction (PPI) network analysis were carried out using the obtained common DEGs. The key modules and hub genes in PPI network were obtained by using the plug-in of Cytoscape software. Subsequently, the hub genes were verified using datasets of NASH (GSE180882) and COVID-19 (GSE150316), and further evaluated by principal component analysis (PCA) and receiver operating characteristic (ROC). Finally, the verified hub genes were analyzed by single-sample gene set enrichment analysis (ssGSEA) and NetworkAnalyst was used for the analysis of transcription factor (TF)-gene interactions, TF-microRNAs (miRNA) coregulatory network, and Protein-chemical Interactions.

**Results:** A total of 120 DEGs between NASH and COVID-19 datasets were obtained, and the PPI network was constructed. Two key modules were obtained via the PPI network, and enrichment analysis of the key modules revealed the common association between NASH and COVID-19. In total, 16 hub genes were obtained by five algorithms, and six of them, namely, Kruppel-like factor 6 (KLF6), early growth response 1 (EGR1), growth arrest and DNA-damage-inducible 45 beta (GADD45B), JUNB, FOS, and FOS-like antigen 1 (FOSL1) were confirmed to be closely related to NASH and COVID-19. Finally, the relationship between hub genes and related pathways was analyzed, and the interaction network of six hub genes was constructed with TFs, miRNAs, and compounds.

**Conclusion:** This study identified six hub genes related to COVID-19 and NASH, providing a new perspective for disease diagnosis and drug development.

## Introduction

COVID-19 is an acute respiratory disease caused by severe acute respiratory syndrome coronavirus 2 (SARS-CoV-2) infection. Given that the virus is highly contagious and can be transmitted through respiratory droplets and close contact, it has been prevalent all over the world and caused serious health problems. Novel coronavirus mainly affects respiratory organs, resulting in upper respiratory symptoms such as dry cough, fever, fatigue, and nasal congestion. Patients with severe COVID-19 rapidly develop acute respiratory distress syndrome, metabolic acidosis, coagulation dysfunction, and multiple organ failure (Price-Haywood et al., 2020). However, COVID-19 is also associated with extrapulmonary manifestations, including muscle and joint pain, loss of smell and taste, ocular conjunctival congestion, diarrhea, rash, and neurological symptoms (Gupta et al., 2020). It has been observed that approximately 14%–53% of COVID-19 patients without pre-existing liver disease suffer from mild to moderate liver injury (Ji et al., 2020; Wang et al., 2020), and that the increased proportion of liver injury in severe COVID-19 cases is significantly higher than that in mild cases (Cai et al., 2020; Huang et al., 2020). In addition, patients with previous liver disease present more severe symptoms and have higher mortality as a result of this viral disease (Su and Hsu, 2021).

Non-alcoholic fatty liver disease (NAFLD) is a common liver disease, which is the manifestation of metabolic syndrome in the liver, including simple steatosis, NASH, and liver cirrhosis. It has become the main cause of adult chronic liver disease (Ludwig et al., 1980). NASH is a severe type of NAFLD, which can lead to liver fibrosis and cirrhosis. The global prevalence of NAFLD is 25%, while that of NASH in NAFLD patients is 59.10% (Younossi et al., 2018), and the prevalence rate is increasing year by year. Patients with NAFLD/NASH have been shown to be exposed to a higher risk of severe COVID-19 disease (Boettler et al., 2020; Su and Kao, 2020). A number of studies have demonstrated the association between the odds of intensive care unit admission and mortality in patients with COVID-19 and pre-existing NAFLD or NASH. In addition, the ratio of medical device use during hospitalization has significantly increased, presenting statistical significance (Hashemi et al., 2020). In addition, a large number of database studies in the United States indicated a strong positive correlation between metabolic syndrome and the risk of COVID-19, and among all the hereby investigated metabolic risk factors, the incidence of NASH and COVID-19 was the strongest (Ghoneim et al., 2020). COVID-19 is still in the epidemic stage, and the number of infections is increasing. Understanding the interaction of the two diseases, NASH and COVID-19, is critical for treating COVID-19 patients with NASH comorbidities.

Herein, the interaction mechanism of NASH and COVID-19 was analyzed using bioinformatics. The expression profile data of NASH and COVID-19 were obtained from the Gene Expression Omnibus (GEO) database. After determining the DEGs shared by NASH and COVID-19, functional annotation, PPI network creation, and key module analysis, as well as identification and validation of hub genes, were performed. Finally, six important hub genes were obtained, ssGSEA was performed on the six hub genes, and protein–chemical, gene–TF, and gene–miRNA interaction networks were constructed. Overall, the hub genes obtained from the analysis provides new insights into the potential molecular mechanism of the coexistence of NASH and COVID-19.

## Result

### Identification of DEGs between COVID-19 and NASH

**FIGURE 1:**
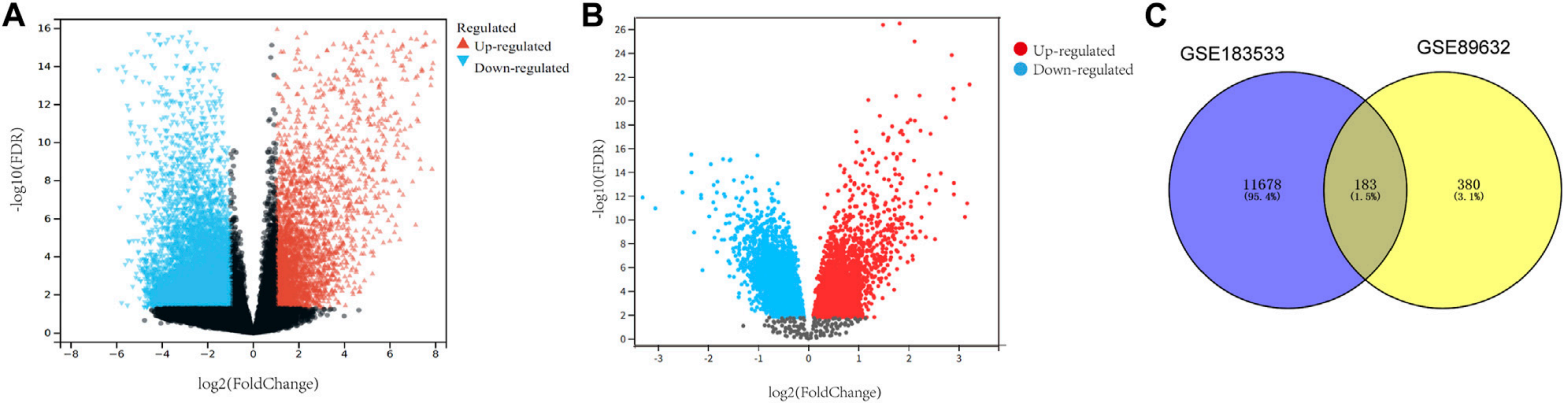
Identification of common DEGs in both NASH and COVID-19. **(A)** Volcano map of the COVID-19 dataset. **(B)** Volcano map of the NASH dataset. **(C)** Venn diagram displaying the shared DEGs among COVID-19 and NASH. DEGs, differentially expressed genes; NASH, non-alcoholic steatohepatitis; COVID-19, coronavirus disease 2019.

In order to study the interaction between NASH and COVID-19, differential analyses for transcriptome datasets for COVID-19 and microarray datasets for NASH were performed. Genes that met the criteria of the false discovery rate (FDR) < 0.05 and |log2FoldChange |≥1 were identified as DEGs, and there were 11,860 DEGs between SARS-CoV-2-infected patients and normal individuals (Figure 1A). Meanwhile, 637 DEGs were observed between NASH patients and normal individuals (Figure 1B). A total of 183 common DEGs were identified after taking the intersection (Figure 1C), and there were 120 DEGs that shared the same expression trend, including 53 upregulated genes and 67 downregulated genes.

### Gene Ontology and Kyoto Encyclopedia of Genes and Genomes analyses

Gene Ontology (GO) analysis focuses on describing the properties of genes or proteins, paying attention to the functions of genes in cells or organisms. Kyoto Encyclopedia of Genes and Genomes (KEGG) analysis focuses on describing the actions or interactions of genes or proteins inside and outside the cell, paying attention to the role of genes in metabolic pathways.

**FIGURE 2:**
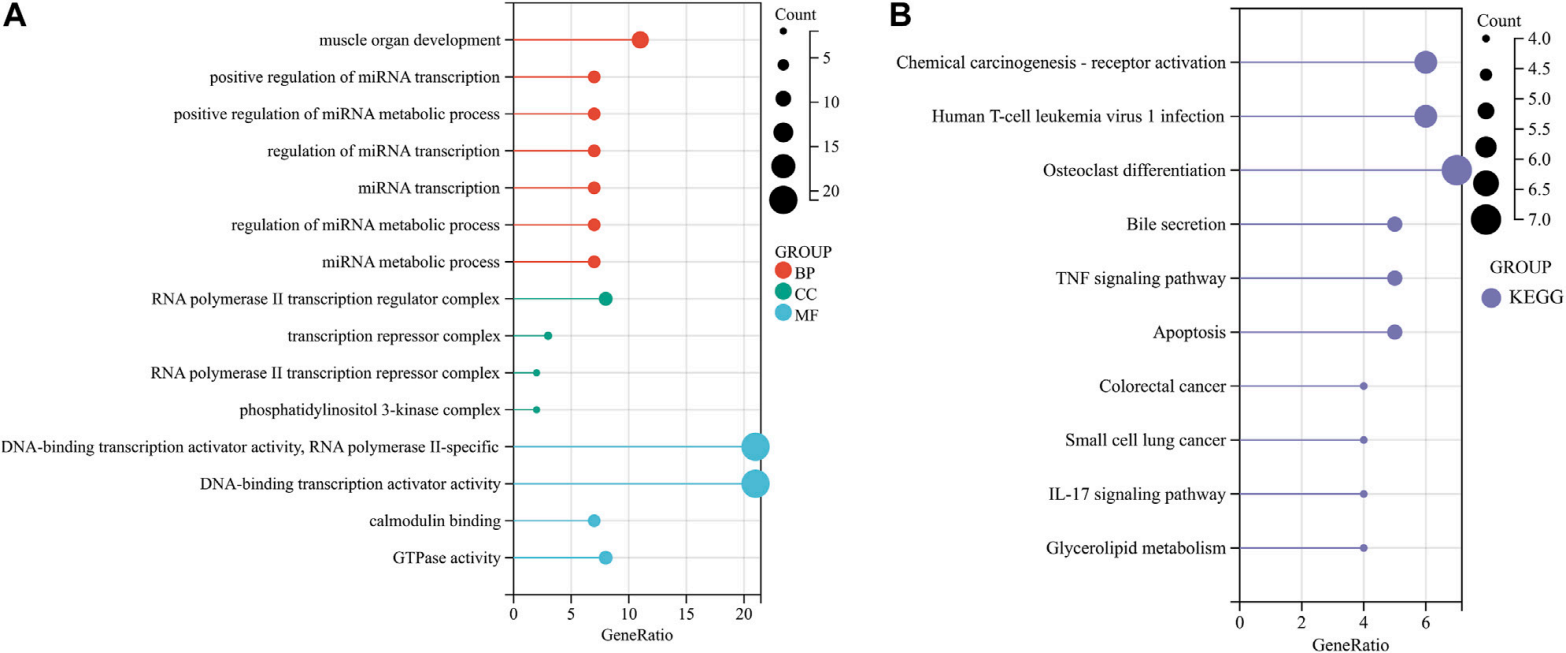
Functional enrichment analysis of common DEGs. **(A, B)** Enrichment analysis results of GO and KEGG pathways. BP, CC, and MF belong to GO analysis. GO, Gene Ontology; KEGG, Kyoto Encyclopedia of Genes and Genomes; BP, biological process; CC, cellular component; MF, molecular function.

In order to further explore the functions and pathways of 120 common DEGs, GO and KEGG enrichment analyses were carried out. GO analysis showed that the genes were mainly enriched in the regulation of miRNA transcription, RNA polymerase II transcription regulator complex, DNA-binding transcription activator activity, and integrated stress response signaling. KEGG analysis showed that the gene was enriched in osteoclast differentiation, bile secretion, TNF signaling pathway, and glycerolipid metabolism pathways (Figures 2A, B).

### PPI network construction and module analysis

**FIGURE 3:**
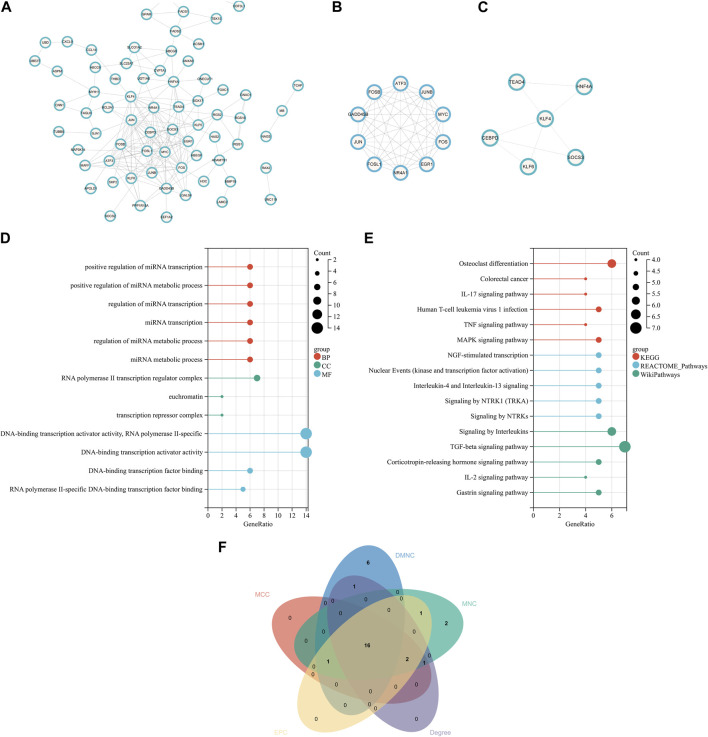
PPI network, analysis of key modules, and identification of hub genes. **(A)** PPI network of the common DEGs. **(B, C)** Key modules of the PPI network. **(D)** GO enrichment analysis results of the modular genes. **(E)** Results of KEGG, Reactome, and WikiPathways enrichment analyses of the modular genes. **(F)** Venn diagram showing that 16 hub genes were obtained by five algorithms. PPI, protein–protein interaction.

In order to explore the interaction between proteins encoded by common DEGs, a PPI network of 120 common DEGs was created using the STRING online website. The network consisted of 116 nodes and 185 edges (Figure 3A). The two most closely related gene modules were obtained using Cytoscape’s MCODE plug-in. Module 1 had 10 nodes and 44 edges, and the score (density multiplied by the number of members) was 9.778 (Figure 3B). Module 2 had six nodes and eight edges, with a score of 3.2 (Figure 3C). In terms of biological function, the genes in the key modules were mainly involved in the regulation of miRNA transcription and metabolic pathways, RNA polymerase II transcription regulator complex, and DNA-binding transcription activator activity (Figure 3D). For the purpose of significant pathway analysis, WikiPathways and Reactome databases were also used alongside the KEGG pathway. The enrichment analysis showed that the genes in the key modules were mainly enriched in inflammatory signaling pathways, for example, IL-17 signaling pathway, TNF signaling pathway, human T-cell leukemia virus 1 infection, TGF-beta signaling pathway, and NGF-stimulated transcription (Figure 3E). Inflammation is a characteristic of NASH and is considered to be the driving force for the progression of the disease to fibrosis, cirrhosis, or HCC. In addition, novel coronavirus can induce systemic inflammation. The inflammatory reaction was closely related to COVID-19 and non-alcoholic hepatitis. Therefore, it was speculated that the inflammation-related pathways involved in the genes in the key modules may play an important role in the occurrence and development of these two diseases. The top enriched shared pathways are summarized in Supplementary Tables S1, S2.

### Hub gene identification

Key genes playing an important role in the coexistence of NASH and COVID-19 were identified using cytoHubba. The top 20 hub genes in the PPI network were obtained by MCC, MNC, DMNC, Degree, and EPC. After the intersection with the online Venn tool, 16 common hub genes were obtained (Figure 3F), which were all downregulated genes. Most of these genes existed in key modules 1 and 2. The top 20 hub genes in five algorithms are listed in Supplementary Table S3.

### Validation of hub genes

**FIGURE 4:**
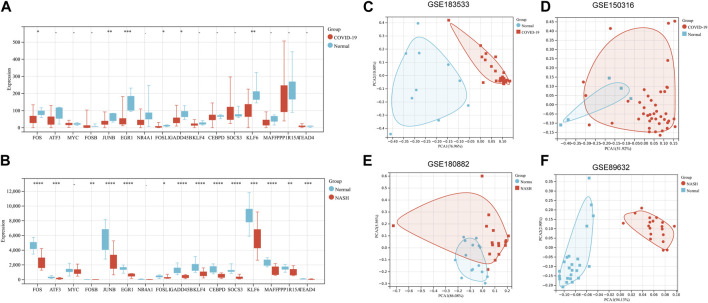
Validation of hub genes. **(A)** Validation of 16 hub genes’ expression in GSE150316 for COVID-19. **(B)** Validation of 16 hub genes’ expression in GSE180882 for NASH. **(C–F)** PCA for six key genes (*FOS*, *FOSL1*, *JUNB*, *KLF6*, *EGR1*, and *GADD45B*) in GSE89632, GSE180882, GSE183533, and GSE150316. *p < 0.05, **p < 0.01, ***p < 0.001. PCA, principal component analysis.

The expression levels of 16 hub genes were verified by GSE150316 for COVID-19 and GSE180882 for NASH. The results showed that compared with normal tissues, six hub genes in COVID-19 and NASH datasets, namely, *FOS*, *JUNB*, *EGR1*, *KLF6*, *FOSL1*, and *GADD45B*, were significantly downregulated (Figures 4A, B). In addition, six hub genes were further identified by PCA, and the results showed that the dimension of these six genes was simplified to two principal components of PC1 and PC2. PC1 and PC2 of two experimental sets and two validation sets were accounted for more than 70% of the total variance. After dimensionality reduction, six genes could clearly distinguish between disease samples and normal samples in four datasets (Figures 4C–F). At the same time, the expression of six hub genes in related cancers was also analyzed. Based on TCGA and GTEx databases, the expression levels of *FOS*, *GADD45B*, and *EGR1* in liver hepatocellular carcinoma (LIHC) and lung adenocarcinoma (LUAD) were lower than those in normal samples. *KLF6* and *JUNB* were downregulated in LUAD and lung squamous cell carcinoma (LUSC) (*p* < 0.05). The results are shown in Supplementary Figures S1A–F.

### Evaluation of hub genes

**FIGURE 5:**
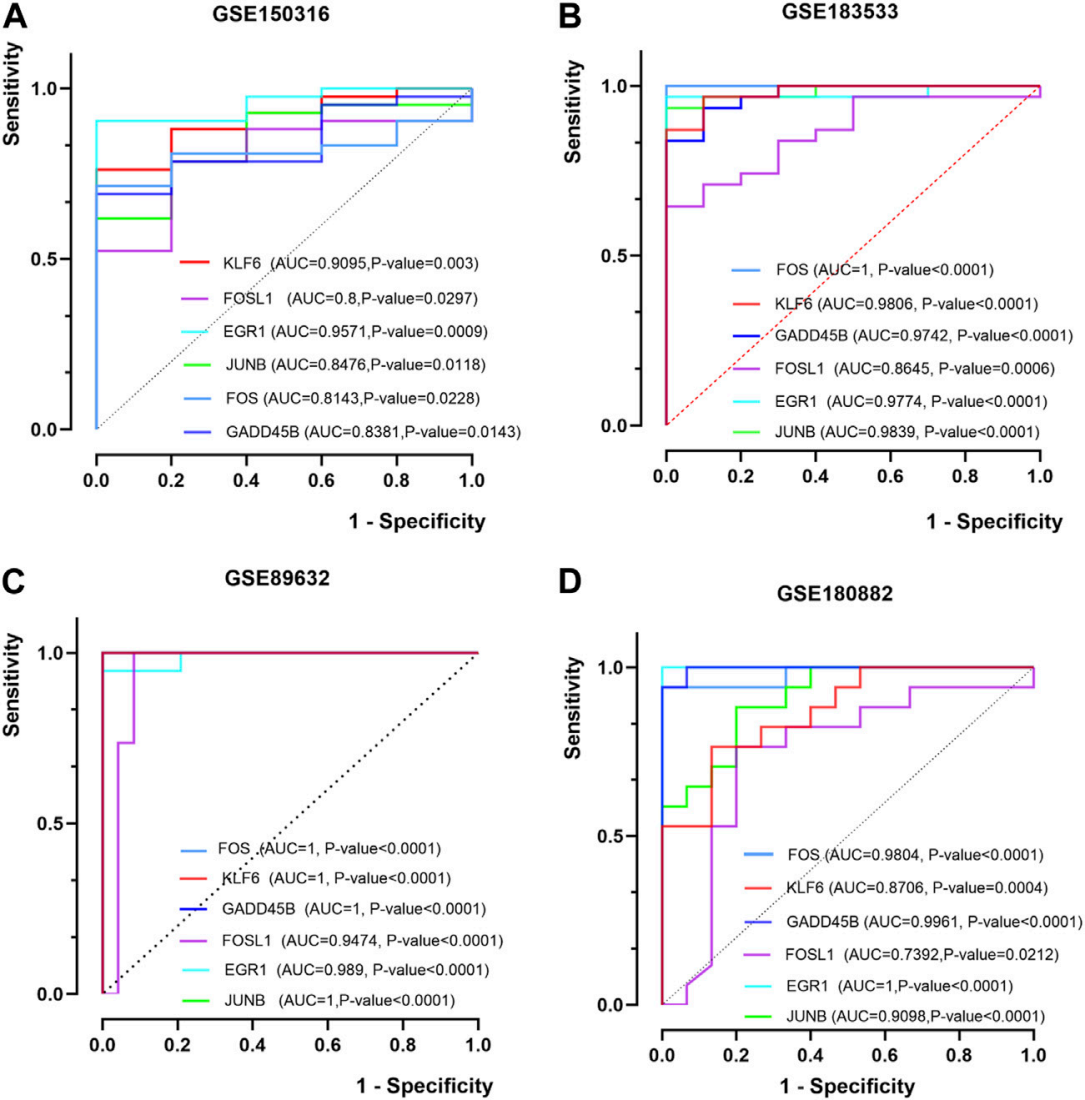
ROC curves of the hub genes. **(A, B)** ROC curves of six hub genes in GSE150316 and GSE183533 for COVID-19. **(C, D)** ROC curves of six hub genes in GSE89632 and GSE180882 for NASH. ROC, receiver operating characteristic; AUC, area under the curve.

The ROC of six hub genes in the datasets (GSE183553, GSE89632, GSE89632, and GSE180882) were drawn to evaluate the prediction accuracy of hub genes. In the four datasets, the area under the curve (AUC) values of five hub genes were more than 0.8 (Figures 5A–D). It was proved that *FOS*, *JUNB*, *EGR1*, *KLF6*, *FOSL1*, and *GADD45B* showed favorable diagnostic value. These results demonstrated the potential of the aforementioned hub genes as biomarkers for the diagnosis of NASH and COVID-19.

### Relationship between hub genes and related pathways

**FIGURE 6:**
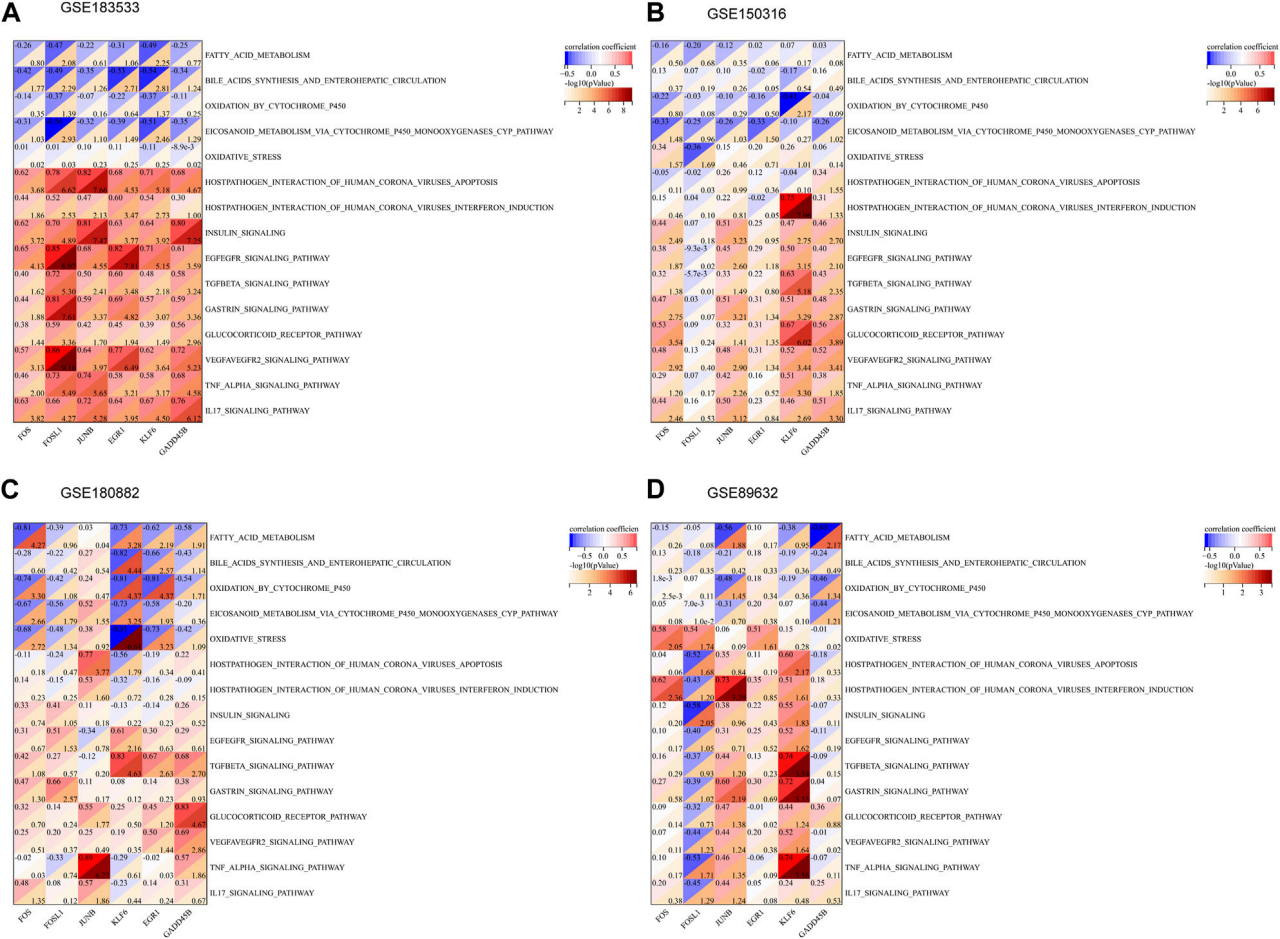
Association between the hub genes and related pathways. **(A–D)** Correlation results of hub genes and 15 pathways in GSE183533, GSE150316, GSE180882, and GSE89632.

Based on the enrichment results of key modules, 30 pathways were selected from KEGG and WikiPathways gene sets, and Spearman’s correlation analysis was used to analyze their relationship with six hub genes (Figures 6A–D). As shown in the figure, *EGR1*, *KLF6*, *FOSL1*, and *FOS* were negatively correlated with fatty acid metabolism, oxidation by cytochrome P450, and eicosanoid metabolism via the cytochrome P450 monooxygenases pathway. *GADD45B* and *KLF6* had a negative correlation with the VEGFA–VEGFR2 signaling pathway, TNF-alpha signaling pathway, and TGF-beta signaling pathway. *JUNB* had a negative correlation with the TNF signaling pathway, gastrin signaling pathway, and IL-17 signaling pathway. In total, 15 of the 30 passageways are shown in Supplementary Figures S2A–D.

### Networks of gene–miRNA and TF–gene interactions

**FIGURE 7:**
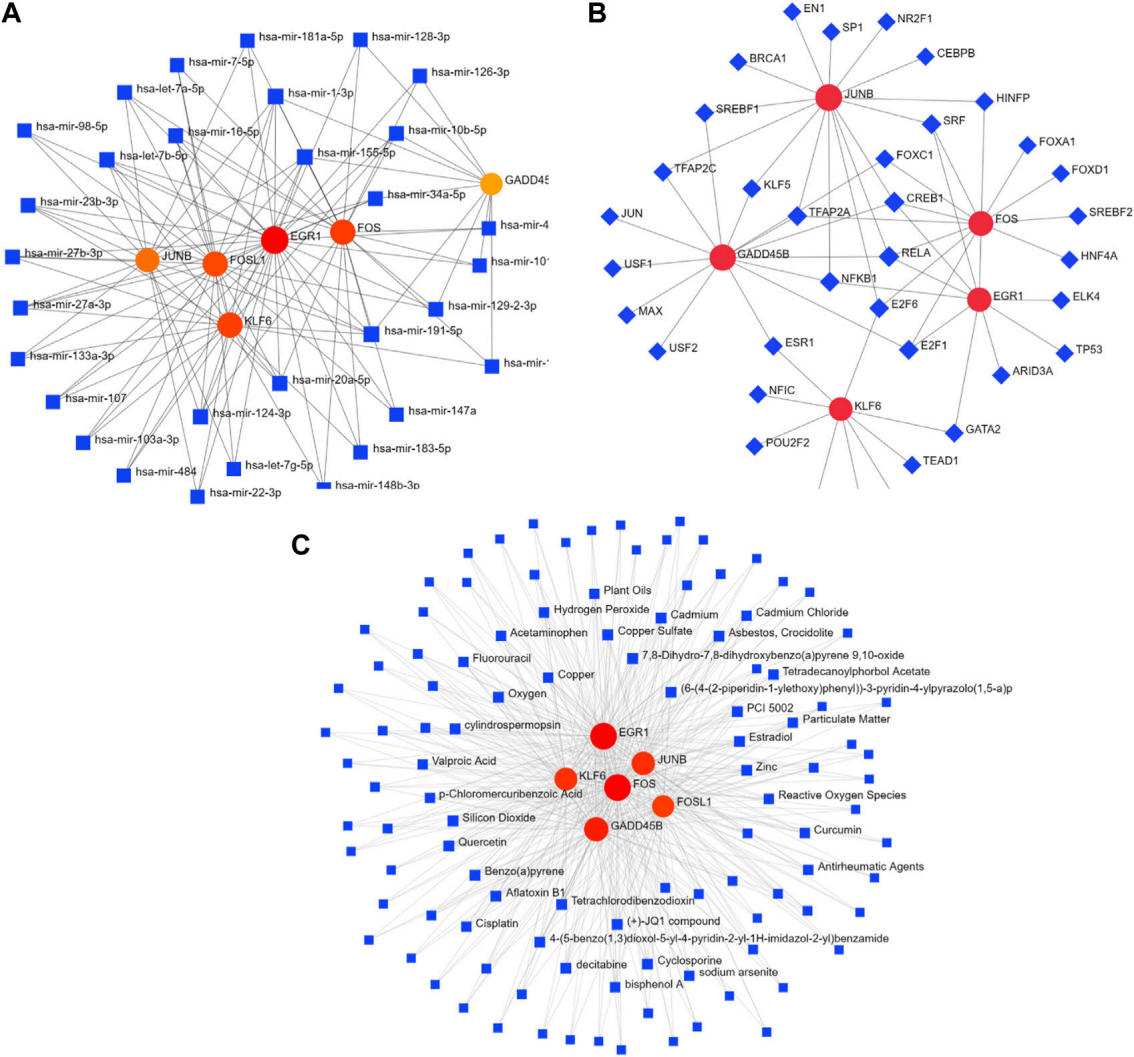
Interaction network of DEGs with TFs, miRNAs, and chemicals. **(A)** Interaction network of TF–DEG. Blue square nodes represent TFs, and gene symbols interacting with TFs are denoted as red circle nodes. **(B)** Interaction network of DEG–miRNA. Blue square nodes are miRNAs, and gene symbols interacting with TFs are denoted as circle nodes. **(C)** Interaction network of protein–chemical. Blue square nodes represent chemicals, and proteins interacting with chemicals are denoted as circle nodes. TFs, transcription factors.

In order to deeply explore the regulatory molecules of hub genes, the gene–miRNA interaction network was constructed, which consisted of 37 nodes and 116 edges. mir-191-5p, mir-155-5p, and mir-1-3p interacted with six hub genes: *FOS*, *JUNB*, *EGR1*, *KLF6*, *FOSL1*, and *GADD45B*. Meanwhile, four hub genes (*EGR1*, *FOS*, *KLF6*, and *FOSL1*) had a higher degree of connection in the network (Figure 7A; Supplementary Table S4). In addition, the TF–gene interaction network comprising 41 nodes and 59 edges was also identified, where TFs interacted with five hub genes: *JUNB*, *GADD45B*, *FOS*, *EGR1*, and *KLF6*. CREB1, RELA, E2F6, and ESR1 were identified as important TFs (Figure 7B; Supplementary Table S5).

### Networks of protein–chemical interactions

Protein–chemical interaction networks help understand the mechanisms of disease occurrence and provide assistance in drug development. Herein, the networks of protein–chemical interactions for hub genes were established, which comprised 114 nodes and 434 edges (Figure 7C; Supplementary Table S6). Aflatoxin B1, oxygen, quercetin, and cyclosporine were the compounds that interacted with all six hub genes.

## Discussion

As SARS-CoV-2 continues to spread worldwide, many studies have shown that patients with NASH are more likely to develop severe COVID-19 after being infected with SARS-CoV-2. In addition, patients with NASH disease will experience increased liver symptoms after being infected with COVID-19 (Bangash et al., 2020; Li and Fan, 2020; Mendez-Sanchez et al., 2020; Rismanbaf and Zarei, 2020). The common symptoms of NASH are obesity, type 2 diabetes, and hyperlipidemia, which are closely related to metabolic syndrome (Eslam et al., 2020). In metabolic abnormalities, expansion of metabolically active fat (high body mass index) can exacerbate chronic inflammatory changes, development of insulin resistance, and fibrosis (Guan et al., 2020; Guo et al., 2020). The detrimental interactions of the complex inflammatory pathways that have long been present in NASH may be dramatically enhanced after infection with novel coronavirus (Portincasa et al., 2020). To improve public understanding of the relationship between COVID-19 and NASH and provide new treatment ideas for patients with COVID-19 and NASH, the possible molecular biological functions and pathways between COVID-19 and NASH were hereby analyzed using bioinformatics.

Herein, 120 common DEGs between COVID-19 and NASH were obtained. The results of GO enrichment analysis showed that DEGs were mainly enriched in the regulation of miRNA transcription, RNA polymerase II transcription regulator complex, and DNA-binding transcription activator activity. Many sources of evidence have suggested the important role of *Homo sapiens* (hsa)-miRNAs in targeting the viral genome, regulating inflammatory signaling pathways, and enhancing the production/signaling of IFNs-I (Xu et al., 2014; Mortazavi-Jahromi et al., 2020a; Mortazavi-Jahromi et al., 2020b; El-Nabi et al., 2020; Guterres et al., 2020; Khan et al., 2020; Nersisyan et al., 2020). It is hypothesized that the immune inflammatory response triggered by COVID-19 affects the development of the disease by influencing the expression level and function of hsa-miRNAs (O'Connell et al., 2007; Ghorpade et al., 2013; Du et al., 2015; Chung et al., 2017). The results of KEGG enrichment analysis showed that DEGs were enriched in bile secretion, TNF signaling pathway, glycerolipid metabolism, and osteoclast differentiation. Generally, the TNF signaling pathway plays an important role in immune-involved inflammatory response and apoptosis (Bradham et al., 1998; Ding and Yin, 2004). The inhibition of cell death pathways mediated by TNF-α and IFN-*γ* to reduce tissue damage and inflammation is an adjuvant therapy for COVID-19 and other inflammatory diseases (Karki et al., 2021). Bile acids matter considerably in insulin sensitivity and metabolic homeostasis. Studies have reported the correlation between bile acid levels and the severity of NASH, as well as the dysregulation of free fatty acid-induced bile acid signaling in NASH (Bechmann et al., 2013). Bile acid derivatives and compounds that affect bile acid-related signaling pathways are considered potentially useful therapeutic agents for NASH (Arab et al., 2017; Gottlieb and Canbay, 2019). Enrichment analysis was performed on genes in key modules with scores greater than 3 in the PPI network. The enrichment results of genes in key modules and DEGs were similar to those in GO analysis. The pathways enriched in KEGG analysis included IL-17 signaling pathway and human T-cell leukemia virus 1 infection. HTLV-1 infection is a systemic inflammatory disease characterized by chronic evolution. Some studies have shown that both SARS-CoV-2 infection and HTLV-1 infection somehow shared similar immunologic properties (Sajjadi et al., 2022). WikiPathways showed that genes were mainly involved in the TGF-beta signaling pathway and network map of the SARS-CoV-2 signaling pathway. The signaling pathway map showed protein–protein interactions and downstream molecular events regulated by SARS-CoV-2, which was expected to contribute to the development of novel targeted therapeutics for COVID-19. Reactome pathways indicated that genes were mainly enriched in NGF-stimulated transcription. The expression of NGF was upregulated in response to liver inflammation or injury. It has been proved that NGF can improve liver fibrosis by regulating HSC cell apoptosis (Oakley et al., 2003). In addition, NGF has also been considered capable of providing a protective mechanism against oxidative damage (Tsai et al., 2014).

Bioinformatic analysis was used to obtain six hub genes associated with NASH and COVID-19 from PPI networks: *FOS*, *JUNB*, *EGR1*, *KLF6*, *FOSL1*, and *GADD45B*. These genes might play an important role in the development of the disease. *GADD45B* is a member of the GADD45 protein family and plays an important role in regulating a variety of cellular functions. *GADD45B* has paradoxical effects: positively, it promotes proliferation, growth and cell survival, playing a dominant role in hepatocytes, while negatively, it inhibits proliferation and stimulates apoptosis, especially in hepatocellular carcinoma. These effects depend on the cellular environment (Tianand and Locker, 2013). Studies have shown that *GADD45B* plays a regulatory role in liver-related metabolic diseases (Fuhrmeister et al., 2016; Cai et al., 2021). *KLF6* is a transcription factor with tumor-suppressive effects. The overexpression of *KLF6* has negative effects on tumor growth and progression; the silence of *KLF6* leads to the increase of tumorigenicity (Ito et al., 2004; Sangodkar et al., 2009; Ahronian et al., 2016; Hsu et al., 2017). Studies have shown that *KLF6* exhibits a cell growth inhibition function by regulating the expression of genes involved in cell cycle, apoptosis, and aging (Narla et al., 2001; Huang et al., 2008; Gao et al., 2017; Sabatino et al., 2019).

*EGR1* is a transcription factor that plays a key role in inflammation and tissue repair (Hoffmann et al., 2008; Trizzino et al., 2021; Woodson and Kehn-Hall, 2022). Studies have shown the importance of *EGR1* in maintaining the hepatic insulin response and claimed that the loss of *EGR1* in hepatocytes leads to hepatic steatosis, which exacerbates the progression of non-alcoholic liver disease. This study revealed a previously unrecognized role of *EGR1* in regulating lipid metabolism (Magee and Zhang, 2018; Li et al., 2019). In addition, the expression of *EGR1* was positively correlated with the expression of Fos gene family, including *FOS*, *FOSB*, *FOSL1*, and *FOSL2*, suggesting that the two immediately expressed gene families might be cross-activated and mutually regulated (Yuan et al., 2022). FOS, FOSL1, and JUNB proteins are subunits of transcription factor activator protein-1 (AP-1)*. JUNB* and *FOS* can combine with *Jun* to form transcription factor complex AP-1. The functions and pathways of these genes in inflammation development and metabolic responses are diverse. For example, studies have shown that FOSL1-deficient animals have less lung injury and a higher survival rate than the control group in experimental models of acute lung injury (Takada et al., 2011; Vaz et al., 2012). Another study has shown that *FOSL1* expression in liver cells protects liver cells from secondary liver injury and inflammation (Hasenfuss et al., 2014). The function and pathway of these genes depend on the stage of the disease and the cellular environment. Herein, KEGG enrichment analysis showed that *FOSL1*, *JUN*, *FOSB*, and *FOS* were enriched in the IL-17 signaling pathway. Reactome pathway enrichment analysis showed that *FOSL1*, *JUNB*, *FOS*, and *EGR1* were enriched in NGF-stimulated transcription and nuclear events (kinase and transcription factor activation). Additionally, the WikiPathways enrichment analysis showed that *JUNB*, *JUN*, *FOS*, and *EGR1* were enriched in the network map of the SARS-CoV-2 signaling pathway and that *FOSL1*, *JUNB*, *FOSB*, and *FOS* were enriched in the corticotropin-releasing hormone signaling pathway.

The correlation of hub genes with some pathways was analyzed by ssGSEA. The results showed that *EGR1*, *KLF6*, *FOSL1*, and *FOS* were negatively correlated with the cytochrome P450 functional pathway, consistent with the increase in cytochrome P450 metabolism in NASH liver-like organs (McCarron et al., 2021). *GADD45B* and *KLF6* were positively correlated with the TGF-β signaling pathway. TGF-β is a multifunctional cytokine with pro-fibrosis, anti-inflammatory, and immunosuppressive effects. In COVID-19 patients, the increased levels of TGF-β may occur to counteract the high inflammatory response (Russell et al., 2020), but it can also slow the recovery of disease *in vivo* by inhibiting the activity of the immune system *in vivo* (Sheng et al., 2015; Ferreira-Gomes et al., 2021). In addition, *JUNB* was hereby found positively correlated with the IL-17 signaling pathway and gastrin signaling pathway. IL-17 is a member of the multifunctional cytokine family. Studies have shown that IL-17 can produce protective and pathological effects *in vivo* (Amatya et al., 2017). The analysis of coronavirus-induced lung diseases has shown that the severity of the disease is positively correlated with the level of IL-17. IL-17 can be used not only as a biomarker of disease severity but also as a potential therapeutic target to mitigate the damage of SARS-CoV-2. However, studies have shown that mice lacking functional IL-17 receptor signaling are more likely to develop secondary pneumonia caused by bacteria after viral infection than wild-type mice (Kudva et al., 2011). The role of IL-17 in the immune system is complex and subtle. Extract-mixed solution can increase the expression of gastrin and motilin to reduce liver fat deposition, protect liver function, and slow down the development of NAFLD (Chen et al., 2013).

In order to further understand the regulatory mechanisms associated with hub genes, TFs and miRNAs related to six hub genes were hereby analyzed. TFs in the network such as CREB1, RELA, E2F1, NFKB1, and TFAP2A play important roles in the regulation of immunity, inflammation, cell proliferation, and apoptosis. The miRNAs that interact more with hub genes are hsa-mir-191-5p, hsa-mir-155-5p, hsa-mir-1-3p, hsa-mir-20a-5p, and hsa-mir-124-3p. Studies have shown that hsa-mir-191-5p regulates mitochondrial function by regulating the level of gene expression (Quiñones-Lombrañan and Blanco, 2015). Mitochondrial dysfunction leads to the formation of reactive oxygen and reactive nitrogen, increased steatosis, and lipid accumulation in hepatocytes (Grattagliano et al., 2012). In addition, hsa-mir-155-5p is involved in the regulation of immune response to SARS-CoV-2, cytokine storm, and antiviral processes (Qi et al., 2021). hsa-mir-124-3p is considered a potential candidate for treating COVID-19 and regulating ACE2 networks (Wicik et al., 2020; Prasad et al., 2021). Sardar et al. (2020) showed that the antiviral miRNAs, such as hsa-mir-1-3p and hsa-mir-20a-5p, were downregulated in the blood of patients with viral respiratory diseases.

Finally, several compounds that interacted with hub genes were identified through NetworkAnalyst. Quercetin is a well-known natural polyphenol with strong antioxidant, anti-inflammatory, immunomodulatory, and antiviral properties. *In vitro* and *in vivo* studies have shown that quercetin can be used as a candidate drug for the treatment of COVID-19 (Di Pierro et al., 2021; Saeedi-Boroujeni and Mahmoudian-Sani, 2021). Decitabine belongs to nucleoside analogs and is known as a broad-spectrum antiviral drug that inhibits viral transcription or replication by inhibiting DNA methylation (Ianevski et al., 2018). Several studies on cell lines have found that decitabine reduces SARS-CoV-2 replication (Zhang et al., 2020; Jang et al., 2021; Zheng et al., 2021). In addition, several clinical trials have been conducted to evaluate the therapeutic efficacy of decitabine in critically ill COVID-19 patients. Meanwhile, zinc is endowed with great significance in enhancing immune function and reducing inflammation (Bonaventura et al., 2015; Maares and Haase, 2016). In elderly subjects, the decrease in circulating zinc concentration is associated with increased levels of cytokines such as IL-6, IL-8, and TNF-α (Mariani et al., 2006; Barnett et al., 2010), making it a qualified candidate as a prophylaxis and adjuvant treatment for COVID-19 in high-risk groups.

In the post-epidemic era, how to provide better medical advice for patients with complications is an essential problem to be solved. In this study, to increase the understanding of the relationship between COVID-19 and NASH, bioinformatic analysis was used to identify key genes in COVID-19 and NASH, analyze gene-related pathways, and identify TFs, miRNAs, and compounds that interact with the genes. It is expected that the study results can provide new ideas for drug development in patients with complications, but the research also has some limitations: (i) the study fails to judge whether the downregulation of hub genes can lead to the deterioration of the disease or has a protective effect on the body. Further study will be carried out if more clinical information can be collected in the future; and (ii) the hub genes identified in the study are calculated and analyzed by bioinformatics and have not been verified in clinical trials or *in vitro* studies.

## Method

### Data source

The datasets used in the study were downloaded from the GEO database (http://www.ncbi. nlm. nih.gov/geo). “NASH” and “COVID-19” were used as keywords for searching the related gene expression dataset. The GSE183533 dataset contains the RNA sequencing results of lung samples from 31 patients with COVID-19 and 10 healthy individuals. Meanwhile, the GSE89632 dataset contains liver biopsy samples from 19 patients with NASH and 24 healthy individuals. The validation dataset includes GSE180882 (NASH) and GSE150316 (COVID-19).

### Identification of DEGs in NASH and COVID-19

By using the transcriptome count data difference analysis tool of the Sangerbox online website (http://vip.sangerbox.com/home.html), the GSE183533 dataset was analyzed using the DESeq2 (version 1.32.0) package in R software. The GEO2R (www.ncbi.nlm.nih.gov/geo/ge2r) online analysis tool was used to perform differential analysis on the GSE89632 dataset. The differential genes between the experimental group and the control group were obtained, and genes that met the criteria of FDR <0.05 and |log2FoldChange |≥1 were identified as DEGs. Common DEGs between NASH and COVID-19 datasets were obtained using the online Venn diagram tool (https://bioinfogp.cnb. csic.es/tools/venny/). An adjusted *p*-value was corrected by the Benjamini–Hochberg procedure.

### Enrichment analysis of DEGs

By using the GO/KEGG analysis tool of the online HIPLOT website (https://hiplot-academic.com/), GO and KEGG analyses were performed using the “clusterProfiler” package in R software to investigate possible functions and pathways in DEGs. GO items included biological process (BP), molecular function (MF), and cellular component (CC).

### Construction of the PPI network and analysis of key modules

The PPI network of DEGs with a score (medium confidence) larger than 0.4 was constructed by the interaction gene search database search tool (STRING) (https://cn.string-db.org/) and visualized using Cytoscape 3.9.0. The plug-in MCODE of Cytoscape was used to identify important modules in the PPI network. The criteria were set as follows: Degree Cutoff Module = 2, Node Score Cutoff = 0.2, K-Core = 2, and Max Depth = 100. In addition, to analyze the functional pathways of key modules, GO, KEGG, Reactome and WikiPathways enrichment analyses of genes in key modules (clusters with scores >3) were performed on the online HIPLOT website. Enriched terms with adjusted *p* < 0.05 were considered significant.

### Identification of hub genes

Hub genes of the PPI network were identified by five algorithms of Cytoscape plug-in cytoHubba: MCC, DMNC, MNC, Degree, and EPC. Intersection genes ranked in the top 20 among the five algorithms were identified as the hub genes.

### Verification of hub genes by external datasets

The expression levels of the identified hub genes were verified in GSE150316 and GSE180882. The samples of the NASH dataset (GEO accession ID: GSE180882) were live liver tissue, containing 18 samples of NASH and 15 healthy samples. A total of 42 lung tissue samples infected with SARS-CoV-2 and five healthy lung tissue samples were selected from the GSE150316 dataset. The non-parametric test was conducted to compare the two groups of data. *p* < 0.05 was considered to be significant.

### Verification of hub genes by PCA

The PCA visualization tool of the Sangerbox online website was used to test the ability of hub genes to distinguish different samples using the prcomp function for dimensionality reduction analysis.

### Evaluation of hub genes by the ROC curve

The ROC curves were plotted using GraphPad Prism 8.0 software to evaluate the prediction accuracy of hub genes.

### Single-sample gene set enrichment analysis

The enrichment scores of samples in the c2.cp. kegg.v7.4. and c2.cp. wikipathways.v7.4 gene sets were calculated by the gene set variation analysis (GSVA) tool of the Sangerbox online website (https://vip. Sangerbox. com/home.html). The correlations between six hub genes and pathways were determined by Spearman’s correlation analysis.

### TF–DEG and DEG–miRNA interactions

The NetworkAnalyst platform (https://www.networkanalyst.ca/NetworkAnalyst/) was used to find the TFs interacting with the hub genes from the JASPAR database. The miRNAs interacting with the hub genes were found from TarBase v8.0. The TF–DEG interaction network and DEG–miRNA interaction network were determined.

### Protein–chemical interactions

Compounds interacting with the hub genes were identified from The Comparative Toxicogenomics Database (CTD) using the NetworkAnalyst platform, and the protein–chemical interaction network was correspondingly constructed. The degree cutoff was set to 2.

## Data Availability

The datasets presented in this study can be found in online repositories. The names of the repository/repositories and accession number(s) can be found in the article/Supplementary Material.
