# Supplementary material for: Bioinformatic and systems biology approach revealing the shared genes and molecular mechanisms between COVID-19 and non-alcoholic hepatitis
Source: Front Mol Biosci. 2023 Jun 19;10:1164220. doi: 10.3389/fmolb.2023.1164220 (PMC10315682; doi:10.3389/fmolb.2023.1164220)
Supplement: Supplementary file 4 [file Table1.DOCX]

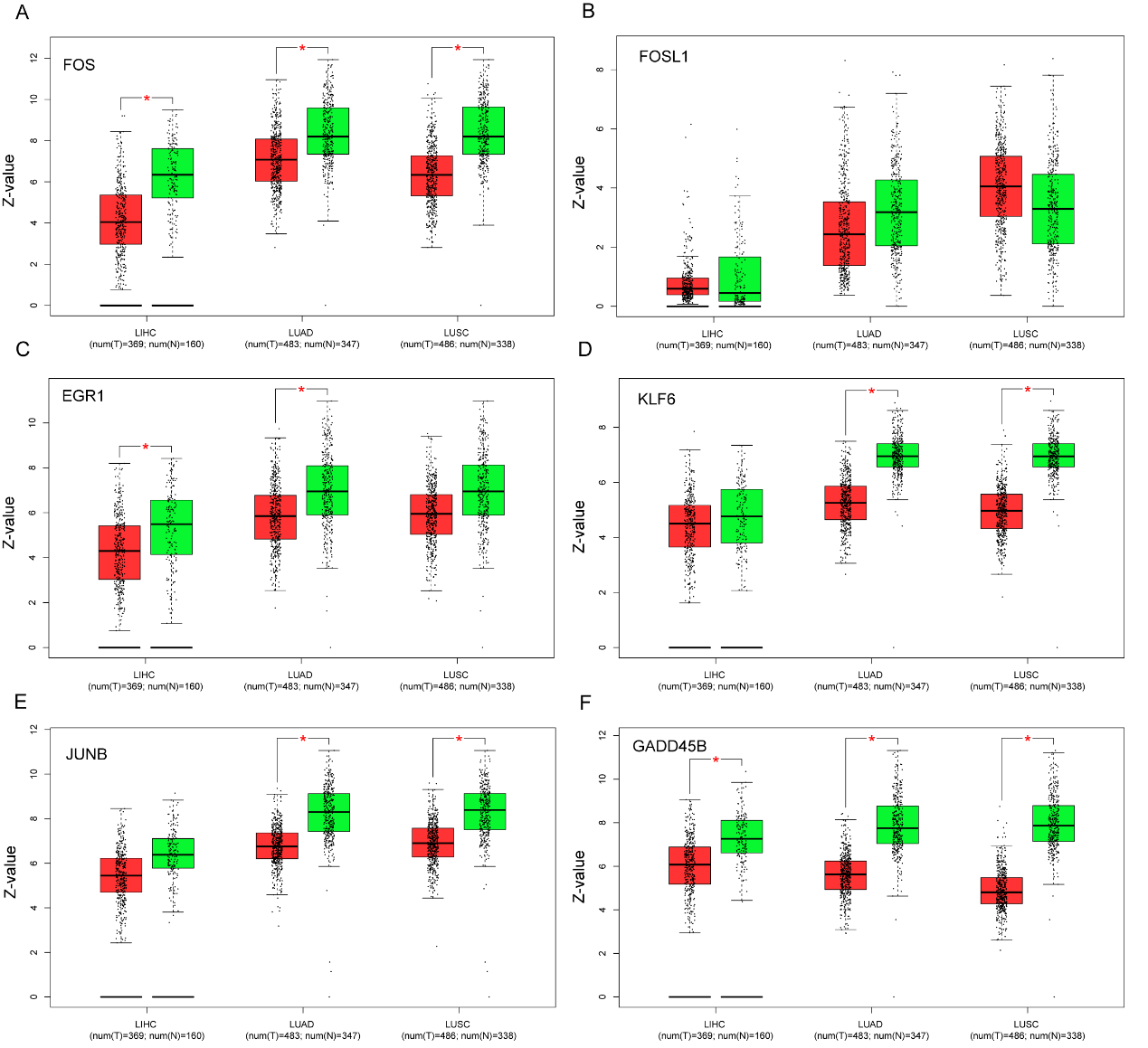


Supplemental Figure 1

Expression level of six hub genes in LIHC, LUAD, LUSC. (A)FOS, (B)FOSL1, (C)EGR1, (D)KLF6, (E)JUNB, (F)GADD45B. *p < 0.05. LIHC, liver hepatocellular carcinoma; LUAD, lung adenocarcinoma; LUSC, lung squamous cell carcinoma.


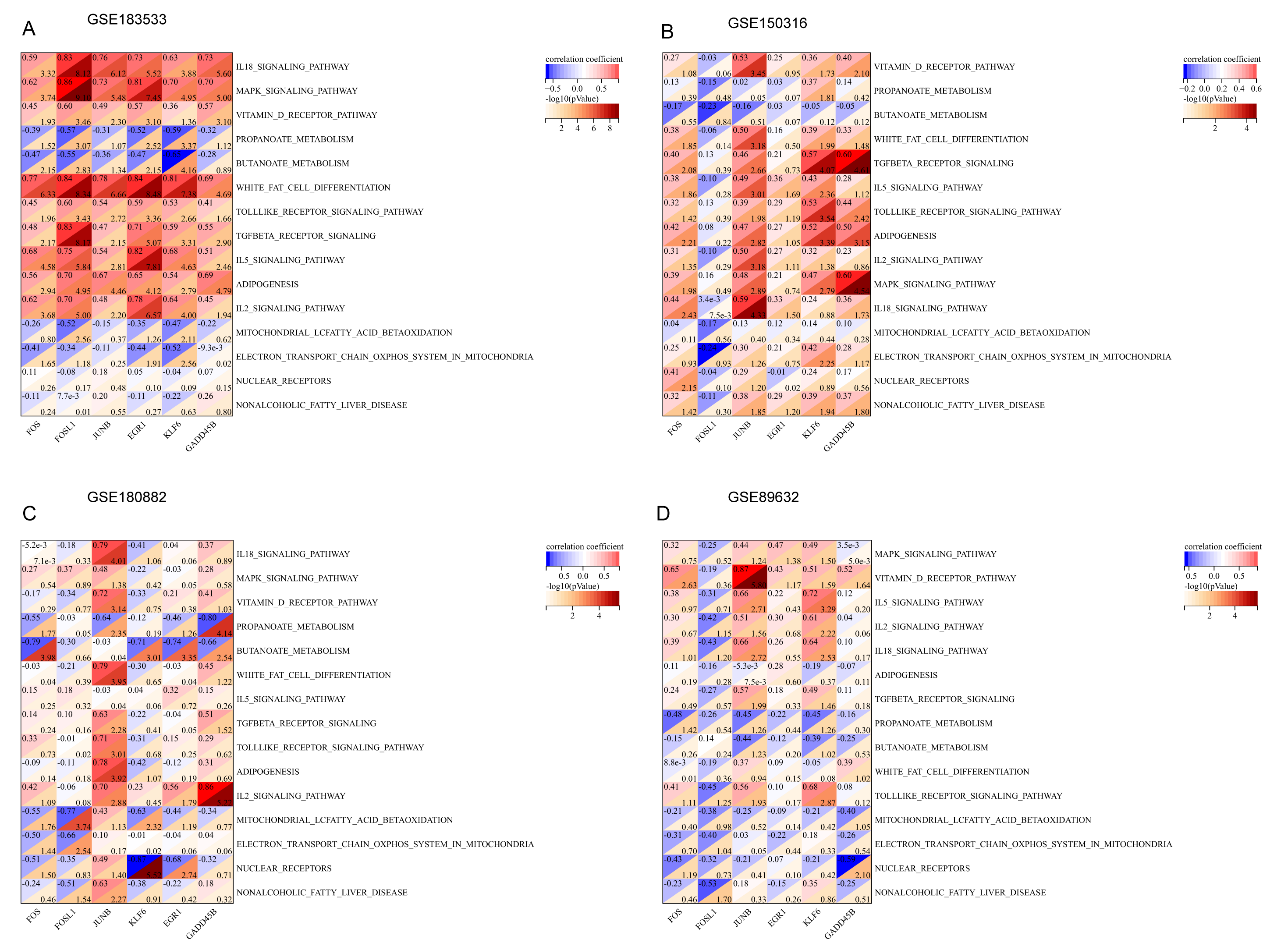


Supplemental Figure 2

Association between the hub genes and related pathways. (A-D) The correlation results of hub genes and another fifteen pathways in GSE183533, GSE150316, GSE180882 and GSE89632.
